# Hyponatremia at the onset of necrotizing enterocolitis is associated with intestinal surgery and higher mortality

**DOI:** 10.1007/s00431-021-04339-x

**Published:** 2021-12-21

**Authors:** Elena Palleri, Veronica Frimmel, Urban Fläring, Marco Bartocci, Tomas Wester

**Affiliations:** 1 Department of Women’s and Children’s Health Karolinska Institute, Stockholm, Sweden; 2 Department of Neonatology, Astrid Lindgren Children’s Hospital Karolinska University Hospital, Solna S3:03, 171 76, Stockholm, Sweden; 3 Department of Pediatric Perioperative Medicine and Intensive Care, Astrid Lindgren Children’s Hospital, Karolinska University Hospital Solna, Stockholm, Sweden; 4 Department of Physiology and Pharmacology, Karolinska Institute, Stockholm, Sweden; 5 Department of Pediatric Surgery, Astrid Lindgren Children’s Hospital, Karolinska University Hospital, Stockholm, Sweden

**Keywords:** Necrotizing enterocolitis, Plasma sodium, Neonatal surgery, Preterm neonates

## Abstract

It has previously been shown that hyponatremia reflects the severity of inflammation in pediatric gastrointestinal diseases. Interpretation of electrolyte disorders is a common, but not well studied challenge in neonatology, especially in the context of early detection of necrotizing enterocolitis and bowel necrosis. The aim of this study was to assess if hyponatremia, or a decrease in plasma sodium level, at the onset of necrotizing enterocolitis (NEC) is associated with intestinal ischemia/necrosis requiring bowel resection and/or NEC-related deaths. This was a retrospective cohort study including patients with verified NEC (Bell’s stage ≥ 2) during the period 2009–2014. Data on plasma sodium 1–3 days before and at the onset of NEC were collected. The exposure was hyponatremia, defined as plasma sodium < 135 mmol/L and a decrease in plasma sodium. Primary outcome was severe NEC, defined as need for intestinal resection due to intestinal ischemia/necrosis and/or NEC-related death within 2 weeks of the onset of NEC. Generalized linear models were applied to analyze the primary outcome and presented as odds ratio. A total of 88 patients with verified NEC were included. Fifty-four (60%) of them had severe NEC. Hyponatremia and a decrease in plasma sodium at onset of NEC were associated with increased odds of severe NEC (OR crude 3.91, 95% CI (1.52–10.04) and 1.19, 95% CI (1.07–1.33), respectively). Also, a sub-analysis, excluding infants with pneumoperitoneum during the NEC episode, showed an increased odds ratio for severe NEC in infants with hyponatremia (OR 23.0, 95% CI (2.78–190.08)).

*Conclusions*: The findings of hyponatremia and/or a sudden decrease in plasma sodium at the onset of NEC are associated with intestinal surgery or death within 2 weeks.

**Table:** 

**What is Known:** *• Clinical deterioration, despite optimal medical treatment, is a relative indication for surgery in infants with necrotizing enterocolitis.* *• Hyponatremia is a common condition in preterm infants from the second week of life.*	
**What is New:** *• Hyponatremia and a decrease in plasma sodium level at the onset of necrotizing enterocolitis are positively associated with need of surgery or death within 2 weeks.* *• In infants with necrotizing enterocolitis, without pneumoperitoneum, where clinical deterioration despite optimal medical treatment is the only indication for surgery, hyponatremia, or a decrease in plasma sodium level can predict the severity of the disease.*

**Supplementary information:**

The online version contains supplementary material available at 10.1007/s00431-021-04339-x.

## Introduction

Necrotizing enterocolitis (NEC) is a severe inflammatory intestinal disease that predominantly affects extremely preterm newborns. Over the last decade, the incidence of NEC in Sweden has increased from 6 to 10% among extremely preterm newborns born before 27 weeks of gestation [1, 2]. When inflammation and intestinal injury are limited, NEC can be treated medically, but if the inflammation progresses to necrosis and bowel perforation, surgery is needed [3]. The need for surgical treatment in NEC patients is a risk factor for adverse neurodevelopmental outcomes, gastrointestinal complications, and death [4, 5]. Bell’s staging system is based on clinical and radiological findings and is still used to assess the severity of the disease [6]. Based on disease presentation, infants with NEC can be classified into two categories: Those who respond to medical treatment and those who require surgical treatment [7]. The identification of infants with progressive disease is difficult and surgical timing tends to be a challenge. Pneumoperitoneum seen on abdominal radiographs is still the only absolute indication for surgical intervention [8, 9]. Since pneumoperitoneum is not always present in neonates with bowel perforation or bowel necrosis [9, 10], deteriorating clinical condition, despite optimal medical management, often becomes a relative indication for surgery [9]. Hyponatremia has been described as one of the metabolic derangements related to clinical deterioration in NEC and other pediatric surgical diseases [11–14], because it seems to reflect the severity of inflammation through the activation of arginine-vasopressin (AVP) resulting in water retention and hyponatremia [12, 15]. Apart from being a very common condition in preterm infants [16], hyponatremia has also been studied as an independent predictor of bowel ischemia [15]. However, it is still not clear if hyponatremia is a predictor of the severity of the disease in infants with NEC. Our hypothesis was that hyponatremia and/or a decrease in plasma sodium (P-Na) concentration at the onset of NEC are positively associated with the presence of ischemic or necrotic bowel and therefore with the severity of the disease. The aim was to assess if hyponatremia and/or a decrease in P-Na concentration at the onset of NEC are positively associated with the severity of disease.

## Material and methods

### Study design

This was a retrospective cohort study. Data were reported according to the Strengthening the Reporting of Observational Studies in Epidemiology (STROBE) checklist [17].

### Study setting and participants

All patients from the Stockholm County who received a NEC diagnosis (P77.9 according to ICD-10) from 1 January 2009, to 31 December 2014, were identified in the Swedish Neonatal Quality Register. Based on established clinical and radiological criteria, the diagnostic accuracy was confirmed using the patients’ case records. A NEC diagnosis was based on the presence of intramural gas/portal gas on abdominal radiographs and/or histological evidence of NEC. Infants with an uncertain diagnosis of NEC (Bell’s stage < II) were not considered to have NEC; hence, they were excluded from the study. The severity of NEC was assessed using Bell’s staging system. Infants with major abdominal malformation or spontaneous intestinal perforation were also excluded from the study. The study population was initially collected for another study analyzing clinical and radiographic findings in NEC [18].

### Data sources

Clinical and demographic data were collected from the case notes. Laboratory data (sodium, creatinine, lactate, glucose) were collected from 3 days prior to NEC onset. Clinical data and data on metabolic status were collected retrospectively and independently by 2 different researchers (EP and VF).

### Exposures

The main exposure was plasma sodium concentration, which was studied in two ways: (1) hyponatremia and (2) the difference in P-Na (ΔNa). Hyponatremia was defined as a plasma sodium (P-Na) < 135 mmol/L at the onset of NEC. ΔNa was defined as the difference in P-Na obtained before NEC onset and at NEC onset. The cut off of 135 mmol/L was also confirmed by a ROC curve (Supplemental Material 1). P-Na was measured by blood gas machine analysis and/or laboratory analysis. In each patient, P-Na was measured with the same type of analysis. NEC onset was defined as the moment when the clinician suspected NEC, obtained blood samples from the infants, and started medical treatment (fasting and antibiotics). P-Na before NEC onset was defined as the closest P-Na value measured (from 24 to 72 h) before NEC onset, when there was no suspicion of NEC. In order to check that this value was representative of a baseline P-Na value for the specific individual, two previous and consecutive P-Na values were checked as well. This value was representative of the P-Na when the patient was clinically stable, when there was no suspicion of NEC and none of the infants had low blood pressure requiring vasoactive support. Patients were excluded if data on metabolic status were not reported. Missing data are reported in the tables.

### Outcomes

Primary outcome was severe NEC, defined as the need for intestinal resection because of bowel necrosis/ischemia and/or NEC-related death within 2 weeks of the onset of NEC. NEC related death was defined as such after reviewing the pathology report from either autopsy or surgical specimen. A composite outcome was chosen because surgery and death are competing risks in NEC patients. A sub-analysis with infants without pneumoperitoneum during the NEC episode was performed for two reasons: (1) to exclude cases with misdiagnosed spontaneous intestinal perforation [19] and (2) because in our setting pneumoperitoneum is an absolute indication for surgery and perfectly predicts the composite outcome.

### Statistical analysis

Patient and clinical characteristics were reported using median and interquartile range or mean and standard deviation for continuous variables, and frequencies and percentages for categorical variables. Mann–Whitney *U* test, Student *T* test, and *chi*^2^ or Fisher exact test were used, when appropriate, to compare clinical characteristics between patients who developed severe NEC and those who did not. Generalized linear models were applied with logit link to analyze the primary outcome and presented as odds ratio with 95% confidence interval (95% CI) of severe NEC. We drew a directed acyclic graph (DAG) to identify possible confounders that may induce non-causal association between plasma sodium concentration at NEC onset and severe NEC. The DAG was used for selection of covariates and is shown in supplemental material 2. Phi coefficient, Spearman correlation, and variance inflation factor (VIF) were used to test for multicollinearity. A *p* value < 0.05 was considered statistically significant. We used the statistical program STATA version 14.2 (StataCorp, College Station, TX USA) to perform the analyses.

### Ethics

Ethical approval was obtained from the Ethical Review Board in Stockholm (No 2017/1237–31 with amendment No 2019–06,289).

## Results

### Participants

**Fig. 1:**
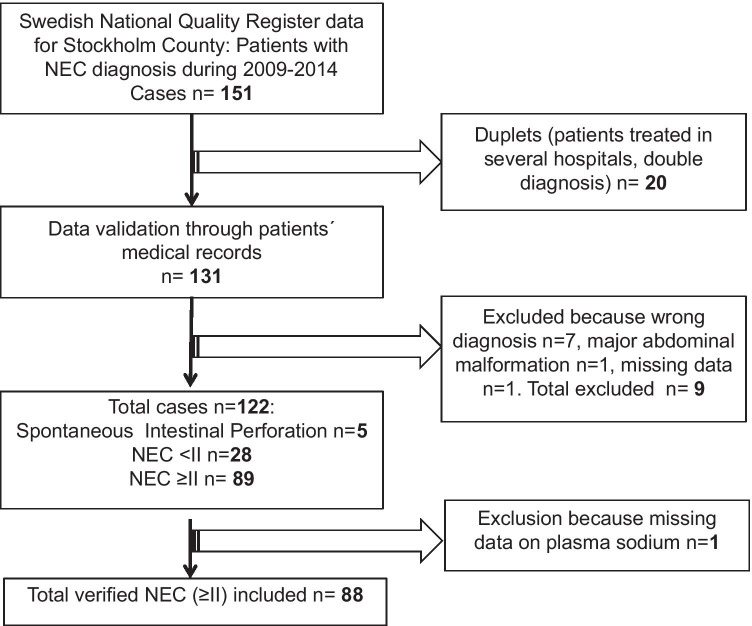
Flow chart, patients

Eighty-nine infants with a verified diagnosis of NEC were identified (Fig. 1). Due to missing P-Na data in one patient, only 88 infants were included in the study. Median gestational age was 26.2 (range 23–38.4) weeks, 60 of 88 were extremely preterm infants. Only two infants were born full-term. Hyponatremia was present in 60 patients at NEC onset, median P-Na 132 (interquartile range (IQR) 128–136). Median P-Na before NEC was 137 (IQR 134–139). Patients’ baseline characteristics are shown in Table 1. Fifty-four infants had severe NEC showing bowel ischemia/necrosis and severe inflammation at the histopathology report: 53 of them underwent surgery and one patient with pneumoperitoneum deteriorated quickly and died before surgery. Only one infant had a drainage inserted because of critical conditions, but underwent surgery shortly after. For infants with severe NEC, median time to bowel resection or death from NEC onset was 1 day (IQR 0–2). NEC-related death within the first 6 months of life was seen in 16 of the infants and among these, 12 died within 2 weeks after the onset of NEC. Only 34 infants did not develop severe NEC. Infants who developed severe NEC had a younger postnatal age at NEC onset than those who did not have severe NEC. Hyponatremia was very common in this cohort; P-Na at NEC onset was lower in the group of infants who developed severe NEC compared to those infants who did not (130 vs 134.5 mmol/l, *p* = 0.0016). P-Na before NEC onset did not differ between the two groups. There was a statistically significant difference in both lactate and platelet counts between the two groups at the onset of NEC. Platelet counts were lower in the severe NEC group and plasma lactate concentration was slightly higher.

**Table 1:** Baseline characteristics in the cohort

	**P-Na < 135 mmol/l (*n* = 60)**	**P-Na** ≥ **135 mmol/l (*n* = 28)**	***p* value**
Severe NEC, *n* (%)	43 (71.7)	11 (39.3)	**0.005**
Gestational age at birth (weeks), median (IQR)	25.92 (24.85–28.86)	26.35 (24.86–29.14)	0.37
Birth weight (g), median (IQR)	810 (678–1162)	820 (702–1191)	0.62
Male, *n* (%)	45 (75)	15 (53.6)	0.05
SGA, *n* (%)	14 (23.3)	4 (14.3)	0.40
Cesarean section, *n* (%)	39 (65.0)	18 (64.3)	1.00
Any IVH, *n* (%)	27 (45.0)	9 (32.1)	0.35
PDA, *n* (%)	45 (75.0)	22 (78.6)	0.79
RDS, *n* (%)	47 (78.33)	22 (78.57)	1.00
Post-natal age at NEC onset (days), mean (SD)	15.3 (1.7)	12.0 (2.1)	0.253
Post menstrual age NEC onset (weeks), median (IQR)	28.93 (26.7–31)	28.07 (26.42–32.57)	0.87
Pneumoperitoneum, *n* (%)	20 (33.3)	10 (35.7)	0.82
Inotropes at NEC onset, *n* (%)	4 (6.7)	1 (3.6)	1.00
P-Na before NEC onset (mmol/L) median (IQR)	135 (133–138)	139 (137–142)	**0.0001**
Missing data, *n* (%)	4 (6.7)	1 (3.5)	
P-Na at NEC onset (mmol/L), median (IQR)	129 (127–132)	137.5 (136–141.5)	**0.000**
Na difference (mmol/L), median (IQR)	5 (3–9.5)	1 (2–3)	**0.000**
Glucose at NEC onset (mmol/l), median (IQR)	10.6 (7.6–14.5)	9.4 (8.2–17.2)	0.99
Missing data, *n* (%)	14 (23.3)	13 (46.4)	
Lactate at NEC onset (mmol/l), median (IQR)	2.3 (1.4–3.5)	1.6 (1.1–2.5)	**0.04**
Missing data, *n* (%)	12 (20)	9 (32.1)	
Platelet count at NEC onset (10^9/L), median (IQR)	104.5 (60–195)	111 (64–187)	0.81
Missing data, *n* (%)	4 (6.7)	2 (7.1)	
Creatinine at NEC onset (µmol/L), median (IQR)	64 (39–80)	59.5 (43.5–69.5)	0.66
Missing data, *n* (%)	4 (6.7)	4 (14.3)	
CRP max during NEC episode (mg/L), median (IQR)	115 (53.5–190)	67.5 (31.5–187)	0.44

*IQR* interquartile range, *SGA* small for gestational age, *IVH* intraventricular hemorrhage, *PDA* patent ductus arteriosus, *CRP* C-reactive protein, *P-Na* plasma sodium, (ΔNa) Sodium difference = P-Na before NEC-onset – P-Na at NEC-onset

### Outcomes

Explanatory variables are shown in Table 2. Univariate analyses are shown in supplemental material 3. No interaction term was found significant between the variables studied. Infants with hyponatremia had higher odds of developing severe NEC compared to those with normal P-Na (crude OR (3.91 95% CI (1.52–10.04), *p* = 0.005). The analysis of decrease in P-Na (ΔNa) as a continuous variable showed that the more the plasma sodium had dropped at NEC onset, the higher the odds of developing severe NEC (crude OR 1.19 95% CI (1.07–1.33), *p* = 0.002). Adjusted ORs are shown in Table 3. A sub-analysis excluding infants with pneumoperitoneum was performed. Hyponatremia at the onset of NEC was present in 95.8% of the infants who developed severe NEC without pneumoperitoneum compared to 50% of the infants who did not develop it (*p* = 0.000). Since extremely preterm newborns more often presented with pre-existing hyponatremia compared to more mature infants (40% vs 21%, *p* = 0.087), a sub-analysis for this group was performed: hyponatremia OR crude (4.27 95% CI (1.34–13.55), *p* = 0.014), ΔNa OR crude (1.22 95% CI (1.05–1.41), *p* = 0.010). In two cases, we were uncertain if the clinical history should have been considered as severe NEC or not. One patient had a known thrombogenic condition and later developed clinical NEC, confirmed radiologically, but with very mild symptoms and low infection markers. This patient died from heart failure 13 days after NEC onset. After reviewing the autopsy report, NEC was not considered to be the cause of this patient’s death, but since it was clinically impossible to know if NEC was somehow responsible for worsening of the heart condition we wanted to control our results considering this infant as severe NEC. Another patient underwent surgery because of NEC without any bowel resection, but had an “open abdomen” for 2 days and required an enterostomy. We performed a sensitivity analysis including these 2 patients as a severe NEC. Even after this analysis, the results were consistent: Hyponatremia OR crude (3.67 95% CI (1.43–9.40), *p* = 0.007), ΔNa, OR crude (1.16 95% CI (1.04–1.29), *p* = 0.005).

**Table 2:** Comparison between infants who developed severe NEC and infants who did not. Explanatory variables

	**Non-severe NEC (34)**	**Severe NEC (54)**	***p* value**
Gestational age at birth (weeks), median (IQR)	26.71 (25.29–29.14)	25.86 (24.71–28.57)	0.263
Age at NEC onset (days), mean (SD)	18.74 (13.33)	11.57 (11.53)	**0.009**
Post-menstrual age at NEC onset (weeks), median (IQR)	29.71 (28.14–32.42)	28.07 (26–30.14)	**0.02**
Pneumoperitoneum, *n* (%)	0 (0)	30 (55.56)	**0.000**
P-Na before NEC onset (mmol/L) median (IQR)	137 (134–139)	136 (134–140)	0.744
Missing data, *n* (%)	1 (3)	4 (7.4)	
P-Na at NEC onset (mmol/L), median (IQR)	134.5 (129–139)	130 (128–134)	**0.016**
P-Na < 135 mmol/L at NEC onset, *n* (%)	17 (50.00)	43 (79.63)	**0.005**
ΔNa (mmol/L), median (IQR)	3 (1–5)	5 (2–9)	**0.003**
Missing data, *n* (%)	1 (3)	4 (7.4)	
Glucose at NEC onset (mmol/l), median (IQR)	10 (7.4–13.7)	10.7 (8.5–15.4)	0.308
Missing data, *n* (%)	15 (44)	12 (22)	
Lactate at NEC onset(mmol/l), median (IQR)	1.5 (1–1.8)	2.5 (1.7–4.2)	**0.0001**
Missing data, *n* (%)	9 (26.5)	12 (22)	
Platelet count at NEC onset (10^9/L), median (IQR)	167 (90–299)	82.5 (49–138.5)	**0.0014**
Missing data, *n* (%)	0 (0)	6 (11.1)	
Creatinine at NEC onset (µmol/L), median (IQR)	48 (34–66)	70 (48–81)	**0.01**
Missing data, *n* (%)	1 (3)	7 (12.9)	
CRP max during NEC episode (mg/L), median (IQR)	115.5 (14–203)	107 (58–181)	0.523

*IQR* interquartile range, *SGA* small for gestational age, *IVH* intraventricular hemorrhage, *PDA* patent ductus arteriosus, *CRP* C-reactive protein, (ΔNa) Sodium difference = P-Na before NEC-onset – P-Na at NEC-onset

**Table 3:** Odds ratio in the NEC patients for severe NEC (generalized linear model analysis, severe NEC as dependent variable)

	**Odds ratio (95% confidence interval)**	***p* value**
Hyponatremia, CRUDE	3.91 (1.52–10.04)	**0.005**
Hyponatremia, adjusted for gestational age, post-natal age at NEC onset, creatinine value	4.75 (1.69–13.6)	**0.004**
Hyponatremia, excluding infants with pneumoperitoneum	23.0 (2.78–190.08)	**0.004**
ΔNa^*^, CRUDE	1.19 (1.07–1.33)	**0.002**
ΔNa^*^, adjusted for corrected gestational age and post-natal age at NEC onset	1.21 (1.07–1.36)	**0.002**
ΔNa^*^, excluding infants with pneumoperitoneum	1.24 (1.06–1.43)	**0.004**

^*^(ΔNa) Sodium difference = Sodium before NEC-onset—Sodium at NEC-onset

Because the cut off of 135 mmol/L can mask the real behavior of the variable plasma Na at NEC onset, an analysis using plasma Na concentration as continuous variable was also performed and it shows the consistency of our results (Supplemental Material 4). For most of the variables, there was no multicollinearity; we tested the covariate with Phi coefficient and Spearman correlation whenever appropriate. VIF was also used to study multicollinearity in the regression models to quantify how much the variance is inflated. VIF was high for lactate and hyponatremia; lactate was not planned to be included in the model according to the DAG.

## Discussion

### Key results

The main finding in this study was that hyponatremia and/or a sudden decrease in plasma sodium level at the onset of NEC were both positively associated with increased odds for need of bowel resection or death. For each mmol/l in plasma sodium decrease at the onset of NEC, the odds for severe NEC increases by almost 20%. Hyponatremia or a sudden decrease in P-Na level could be an early predictor of failure of conservative treatment in infants with NEC.

### Limitations

Considering the small number of patients and, of course, the retrospective design, the findings of this study should be interpreted carefully. On the other hand, patients included had a validated diagnosis of NEC and were consecutive. Of course, the diagnosis of NEC is still difficult and there is no international consensus [20]. Anyhow, in medical NEC, the presence of pneumatosis intestinalis seems to be the radiological sign with the highest likelihood of NEC. This way of validating the NEC diagnosis carried the risk of excluding some infants with mild NEC, but it was unlikely to miss infants with severe NEC (who required surgery or died), because the histopathologic report would confirm the NEC diagnosis. Missing data is a major limitation, especially those on P-Na before NEC onset and plasma glucose at NEC onset. Since we could not assume that missing data were at random, plasma glucose was excluded from the regression model. However, according to our DAG, plasma glucose was not a confounder able to induce a non-causal association between the exposure (P-Na concentration at NEC onset) and odds for severe NEC. We do not have data related to sodium supplementation for each infant; therefore, we studied not only hyponatremia itself at NEC onset but also the decrease in P-Na from a clinically stable state to the onset of NEC. One can argue that supplementation of sodium differed during the days before NEC between infants who developed severe NEC and those who did not. If the diagnosis of NEC was delayed, the P-Na concentration measured before NEC could actually have been analyzed in an infant who already had NEC. This could have masked a decrease in P-Na concentration at the second measurement as a low P-Na could lead the clinician to increase the sodium supplementation. Another limitation of this study is that we do not have data on urinary production, but we used serum creatinine concentration as a proxy for renal function and urinary production in our regression model.

### Interpretation

Tepas et al. [11] has previously described hyponatremia as one of the metabolic derangements to consider when timing surgery in NEC patients, but in their study, hyponatremia was defined as P-Na < 130 mmol/L. The novelty of our results is the association between the decrease in P-Na (and not only hyponatremia itself) and the increased odds of developing severe NEC, especially in infants without pneumoperitoneum. Hyponatremia seemed to be an independent risk factor for severe NEC but there was a covariation between lactate values and P-Na at NEC onset, even though no significant interaction term was found between these two variables. Clinically, hyponatremia is a marker of severe inflammation and an elevated lactate value is just a marker of the same process. On the other hand, there was no strong multicollinearity for ΔNa and lactate value and the interaction term between these two variables was not significant.

The early prediction of surgical NEC may improve outcomes by promoting earlier transfer to surgical centers and earlier surgery, possibly resulting in salvage of viable bowel [21]. Ideally, surgical intervention would occur in infants with irreversible intestinal ischemia prior to intestinal perforation [21, 22]. The prediction of progression of bowel inflammation to necrosis and the identification of NEC infants who ultimately will require surgical intervention is very difficult [22]. When there is radiographic evidence of pneumoperitoneum, it may already be too late, especially in an extremely preterm infant [9, 23]. Clinical deterioration despite optimal medical care often becomes a relative indication for surgical treatment, but the timing is still a challenge [21, 24]. Early surgery should not be considered a failure. On the contrary, it could rather be considered an advantage if infants with NEC undergo surgery before the bowel perforation occurs. This approach can potentially have a favorable impact on morbidity and mortality.

Since hyponatremia is a common condition in preterm infants from the second week of life, many of them are given daily sodium supplement. Obviously, the amount of given sodium supplement will affect the P-Na [16]. Although there is no consensus on the definition of hyponatremia among preterm infants, we chose the cut off of 135 mmol/L as reported in adult literature [25] since it is still the most widely accepted [26]. Considering how common hyponatremia is, our suggestion is to focus not only on one single data P-Na value at NEC onset but to also take the trend of the P-Na into account when assessing patients at risk to develop severe NEC. Hyponatremia has been studied not only as a predictor of tissue ischemia in different surgical conditions such as pediatric small bowel volvulus [27], ischemic bowel in small bowel obstruction [15], incarcerated hernias [28], and perforated appendicitis [12, 29], but also as a predictor of the severity of the disease in infants with bronchiolitis [30].

The exact etiology of hyponatremia in surgical conditions is not well understood. Both hypovolemia and severe inflammation could result in the activation of arginine-vasopressin (AVP) production resulting in water retention and hyponatremia [12, 15]. Acute non-osmotic stimuli, such as surgical trauma, may induce AVP release, which has been supported by a lack of a significant association between AVP and plasma osmolarity [31]. Recent studies show that proinflammatory cytokines may regulate AVP secretion and the development of hyponatremia [32]. P-Na is easily analyzed, often a bedside analysis, available around the clock in all kinds of neonatal units. To know that a larger decrease in P-Na corresponds to increased odds of severe NEC at NEC onset can be helpful in the clinical management of NEC infants, especially when the decision to operate or not is unclear and transfer to another hospital might be needed. This is true even for extremely preterm infants where hyponatremia is very common. The decision to operate is straightforward for infants who present with pneumoperitoneum. However, for infants without pneumoperitoneum, the decision is by far less easy. For these infants, we believe that an extra severity marker such as hyponatremia could be helpful.

### Generalizability

Our study cohort could be considered population-based, as the author’s institution is the only center providing tertiary pediatric surgery in the region and the data could be considered generalizable. On the other hand, the generalizability to a broader patient population is limited by just including a population from one single center. Large 95% CIs were shown in some of the analyses, possibly signaling imprecise measurements. A prospective validation of these findings in a larger and prospective cohort would be valuable.

## Conclusion

Infants who present with hyponatremia and/or a sudden decrease in P-Na at the onset of NEC are more likely to require intestinal surgery or to die within 2 weeks. Hyponatremia or a decrease in P-Na should not be considered a cause of NEC or bowel necrosis, but a marker of a biochemical and physiological derangement that leads to severe bowel inflammation and necrosis.

## Supplementary information

Below is the link to the electronic supplementary material.

Supplementary file1 (DOCX 166 kb)

Supplementary file2 (DOCX 273 kb)

Supplementary file3 (DOCX 15 kb)

Supplementary file4 (DOCX 13 kb)

## Data Availability

Data are available upon request.

## Abbreviations

AVP: Arginine-vasopressin
DAG: Directed acyclic graph
IQR: Interquartile range
NEC: Necrotizing enterocolitis
P-Na: Plasma sodium
OR: Odds ratio
VIF: Variance inflation factor
ΔNa: Difference in plasma sodium
95% CI: 95% Confidence interval
