# Supplementary material for: Hyponatremia at the onset of necrotizing enterocolitis is associated with intestinal surgery and higher mortality
Source: Eur J Pediatr. 2021 Dec 21;181(4):1557–65. doi: 10.1007/s00431-021-04339-x (PMC8964626; doi:10.1007/s00431-021-04339-x)

**Supplemental material 1**

Receiving Operating Characteristic curve, plasma sodium value (mmol/L) at NEC onset.


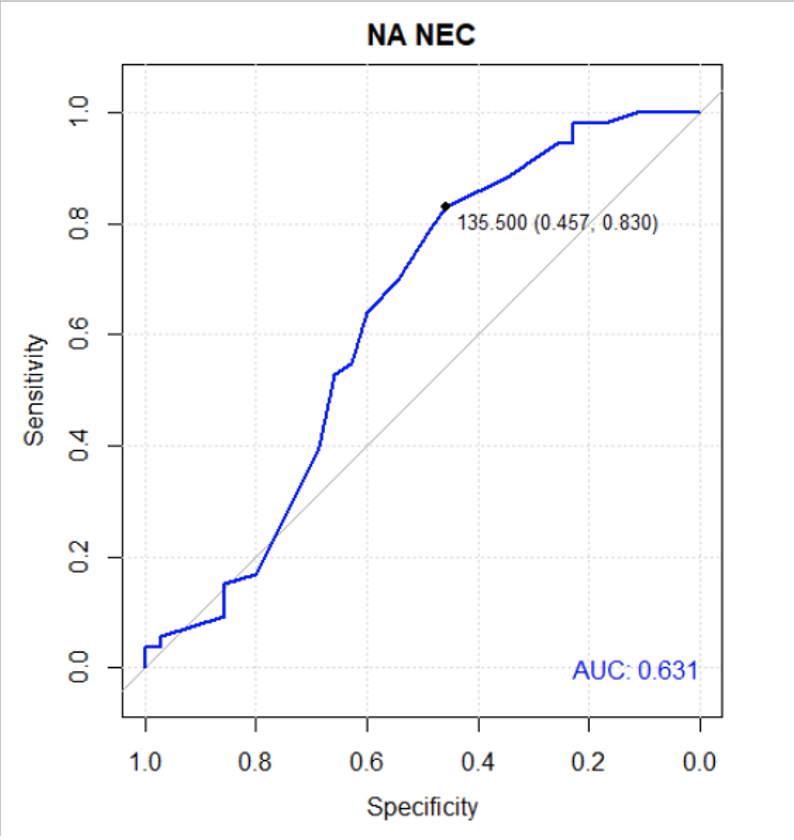

Supplement: Supplementary file 1 — Supplementary file1 (DOCX 166 kb) [file 431_2021_4339_MOESM1_ESM.docx]
