# Supplementary material for: Hyponatremia at the onset of necrotizing enterocolitis is associated with intestinal surgery and higher mortality
Source: Eur J Pediatr. 2021 Dec 21;181(4):1557–65. doi: 10.1007/s00431-021-04339-x (PMC8964626; doi:10.1007/s00431-021-04339-x)
Supplement: Supplementary file 2 — Supplementary file2 (DOCX 273 kb) [file 431_2021_4339_MOESM2_ESM.docx]

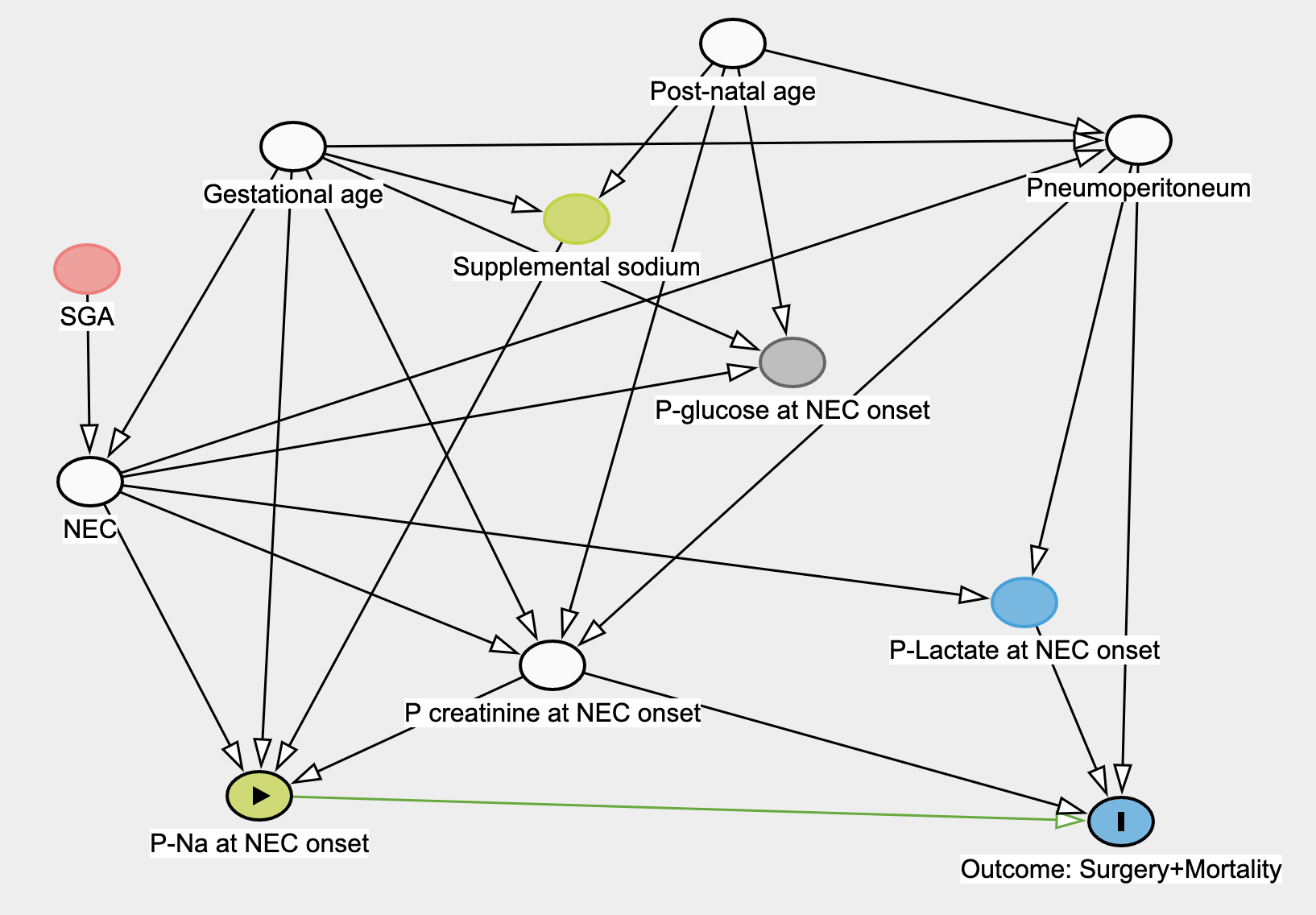


**Directed Acyclic Graphs (DAGs)** are graphs that simplify the interpretation in causal inference. We used it to identify possible confounders that may induce non-causal association between plasma sodium concentration at NEC onset and our outcome (mortality +bowel resection). Each arrow represents a causal influence. In white are the confounders that could induce a non-causal association between hyponatremia and our outcome according to the DAG.
