# Supplementary material for: Hyponatremia at the onset of necrotizing enterocolitis is associated with intestinal surgery and higher mortality
Source: Eur J Pediatr. 2021 Dec 21;181(4):1557–65. doi: 10.1007/s00431-021-04339-x (PMC8964626; doi:10.1007/s00431-021-04339-x)
Supplement: Supplementary file 3 — Supplementary file3 (DOCX 15 kb) [file 431_2021_4339_MOESM3_ESM.docx]

**Supplemental material 3**

**Univariate analyses:**

**Odds ratio for severe NEC** (generalized linear model analysis, severe NEC as dependent variable).

|  | **Odds Ratio** | **p value** |
| --- | --- | --- |
| Hyponatremia, Na<135 at NEC onset | 3.91 (1.52-10.04) | **0.005** |
| Na at NEC onset, as continuous variable | 0.89 (0.82-0.97) | **0.008** |
| *ΔNa | 1.19(1.07-1.33) | **0.002** |
| Gestational age | 0.96(0.85-1.09) | 0.620 |
| Birth weight | 1.03(0.48-2.18) | 0.944 |
| SGA | 0.74(0.26-2.10) | 0.571 |
| Gender male | 0.70(0.47-3.04) | 0.701 |
| Patent ductus arteriosus | 1.26(0.46-4.31) | 0.649 |
| Postnatal age | 0.95(0.92-0.99) | **0.013** |
| C-reactive protein | 1.00(0.99-1.00) | 0.595 |
| Creatinine value | 1.01(0.99-1.02) | 0.420 |
| # Lactate value | 2.67 (1.37-5.22) | **0.004** |
| #Glucose value | 1.04 (0.95-1.14) | 0.356 |
| Vasoactive drugs at diagnosis | 0.94 (0.15-5.94) | 0.949 |

*ΔNa= Na at steady state- Na at NEC onset

# >20% missing values
