# Supplementary material for: Hyponatremia at the onset of necrotizing enterocolitis is associated with intestinal surgery and higher mortality
Source: Eur J Pediatr. 2021 Dec 21;181(4):1557–65. doi: 10.1007/s00431-021-04339-x (PMC8964626; doi:10.1007/s00431-021-04339-x)
Supplement: Supplementary file 4 — Supplementary file4 (DOCX 13 kb) [file 431_2021_4339_MOESM4_ESM.docx]

**Supplemental material 4:**

**Odds ratio in the NEC patients for severe NEC** (generalized linear model analysis, severe NEC as dependent variable). Using plasma sodium (Na) as continuous variable

|  | **ODDS RATIO (95% CONFIDENCE INTERVAL)** | **p-value** |
| --- | --- | --- |
| Na, CRUDE | 0.89 (0.82-0.97) | **0.008** |
| Na, adjusted for gestational age, post-natal age at NEC onset, creatinine value | 0.87(0.79-0.96) | **0.005** |
| Na, excluding infants with pneumoperitoneum | 0.84(0.75-0.95) | **0.006** |
